# Protective humoral immunity in SARS-CoV-2 infected pediatric patients

**DOI:** 10.1038/s41423-020-0438-3

**Published:** 2020-05-07

**Authors:** Yaguang Zhang, Jin Xu, Ran Jia, Chunyan Yi, Wangpeng Gu, Pengcheng Liu, Xinran Dong, Hao Zhou, Bo Shang, Shipeng Cheng, Xiaoyu Sun, Jing Ye, Xuezhen Li, Jia Zhang, Zhiyang Ling, Liyan Ma, Bingbing Wu, Mei Zeng, Wenhao Zhou, Bing Sun

**Affiliations:** 1 State Key Laboratory of Cell Biology, CAS Center for Excellence in Molecular Cell Science, Shanghai Institute of Biochemistry and Cell Biology, Chinese Academy of Sciences, University of Chinese Academy of Sciences, 320 Yueyang Road, Shanghai, 200031 China; 2 Children’s Hospital of Fudan University, 399 Wanyuan Road, Shanghai, 201102 China; 3 Shanghai Kehua Bio-Engineering Co., Ltd.1189 Qinzhou Road, Shanghai, 200233 China

**Keywords:** Infection, Antibodies

After the rapid spread of SARS-CoV-2 in Wuhan, China, at the beginning of 2020, about 1.5 million confirmed cases and over 80,000 deaths have been reported around 200 countries and territories all over the world and the number continues to increase. However, we still have limited knowledge of this new coronavirus, especially the interaction between SARS-CoV-2 and our immune system. In contrast with infected adults, the children have received more attention because of the lower infection rates and milder symptoms.^1^ Less than 1% of infected cases were aged 10 years or younger.^2^ Only 3.5% of SARS-COV-2 infected children had lymphocytopenia.^1^ It has been reported, coronaviruses, including SARS-CoV, MERS-CoV, and SARS-CoV-2, seem to cause fewer symptoms and less severe disease in children compared with adults.^3^ This phenomenon may be related to the differences in the immune responses against the infection of coronaviruses between children and adults. Here, we reported the characteristics of immune response after the SARS-CoV-2 attack in children and found that there is a protective humoral immunity in infected children, in which memory B cells and S-protein specific Abs against the SARS-CoV-2 have been detected. Our observation presents one possible explanation for the milder symptoms in children after exposure to SARS-CoV-2.

We analyzed the T/B lymphocytes in PBMC and the production of antibodies in serum from confirmed cases in pediatrics. The respiratory samples obtained from six patients were all tested positive by RT-PCR for SARS-CoV-2. A cycle threshold value less than 35 was defined as a positive test. Mild cough and sore throat were common symptoms at disease onset among these six patients. None of the patients had diarrhea or dyspnea during illness. The body temperature of three patients was below 38 degrees at disease onset. Chest X-ray showed no pneumonia among three patients. The detailed clinical and epidemiological features of patient 1/2/3/4/5 have been reported previously.^4^ Patient 6 was admitted to Children’s Hospital of Fudan University on 6 Feb and started with mild symptoms as cough and sore throat without fever and other symptoms. Chest X-ray showed no pneumonia. She got infected by SARS-CoV-2 from the household but the interval between symptom onset and exposure to index case is unclear. All patients presented with mild respiratory infections and have been discharged. Informed consent was obtained from the parents or guardians of the patients infected and uninfected with SARS-CoV-2 for the publication of their clinical data. Ethical approval was provided by the Hospital Ethics Committee. Our results showed that pediatric patients had more active B-cell immune responses than uninfected children and obvious antigen-specific antibody production within 2–3 weeks after illness onset. The experiments demonstrated that the neutralizing antibody against the Spike protein of SARS-CoV-2 was detected. This result indicates that there is a protective humoral immunity in children after the SARS-CoV-2 attack.

To have a signature picture of immune responses following the SARS-CoV-2 infection, the RNA prepared from peripheral blood mononuclear cells (PBMC) from a SARS-CoV-2 infected pediatric case and an uninfected control were subjected to RNA sequencing using for Illumina HiSeq™ 2000. An immune system-related GO category enrichment analysis was performed to gain insights into the biological roles of the differential expression genes. We found that B cell-related GO terms were significantly enriched and top of 14 main immune response-related GO categories (S-Fig. 1a). Groups of the differential expression genes were highly enriched in infected case, including mature B-cell differentiation involved in immune response (GO:0002322), positive regulation of humoral immune response (GO:0002922), B-cell activation involved in immune response (GO:0002312) and humoral immune response mediated by circulating immunoglobulin (GO:0002925) (Fig. 1a). The RNA-seq profiling indicates that there is an enhanced humoral immune response responding to SARS-CoV-2 infection in children.

**Fig. 1:**
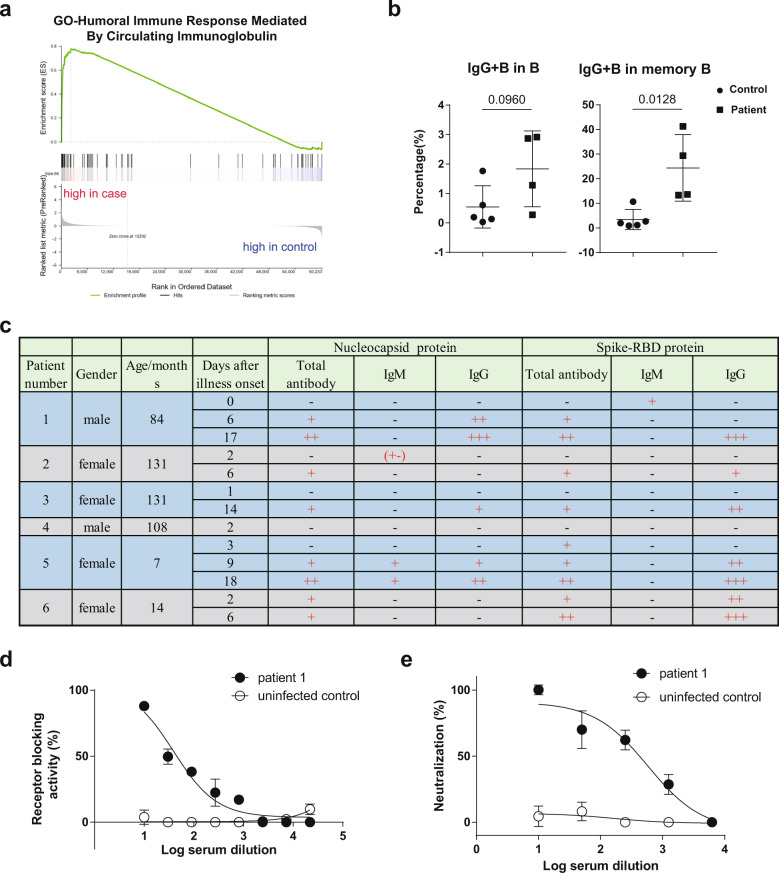
Protective humoral immunity in SARS-CoV-2 infected pediatric patients. **a** Humoral immune response mediated by circulating immunoglobulin (GO:0002925); **b** Percentage of IgG+ B cells in CD19+ B cells and CD27+ memory B cells. The statistical method is the Student’s *t* test. **c** Antibodies detection for nucleocapsid protein and receptor binding domain of Spike of SARS-CoV-2. +: positive, −: negative; (+−): weak negative. **d** Receptor-blocking activity of serum from an infected case and uninfected control. **e** Pseudovirus neutralizing assay of serum from an infected case and uninfected control. The statistical method is the Student’s *t* test

Flow cytometry analysis was performed to analyze T and B cells from four infected pediatric cases and five uninfected controls. The controls were patients hospitalized during the same period without SARS-CoV-2 infection. The T cell gating strategy was shown in S-Fig. 2a. The previous report^4^ showed that the white blood cell count (median: 7.35 × 10^9^/L; normal range: 3.9–9.9 × 10^9^/L) and lymphocyte count (median: 3.25 × 10^9^/L; normal range: 1.2–4.0 × 10^9^/L) were normal in these infected pediatric cases, which was different from the lymphocytopenia in infected adults.^5^ Similar to the unchanged lymphocyte count, the percentage of CD3+, CD4+, and CD8+ T cells between infected and uninfected cases were comparable (S-Fig. 2b). Expression of a chemokine receptor CCR7, in combination with the naive cell marker CD45RA, has been shown to discriminate naïve T cell (NT: CD45RA+CCR7+) and central memory T (TCM: CD45RA−CCR7+) from effector memory T (TEM: CD45RA−CCR7−) and CD45RA+ effector memory T (TET-RA: CD45RA+CCR7−) subsets. We investigated CD45RA and CCR7 expression in CD4+ and CD8+T cells and there were no differences between four infected pediatric cases and five uninfected controls (S-Fig. 2c, 2d).

Due to a more effective humoral immune response in an infected case (S-Fig. 1), B-cell subsets were investigated (S-Fig. 3a). Although the percentage of CD19+ total B cells, IgD+ naive B cells in total B cells and CD27+ memory B cells in total B cells were comparable between infected and uninfected cases (S-Fig. 3b, 3c), the percentage of IgG+ B cells in total B cells were slightly higher in infected cases. More importantly, the percentage of IgG+ B cells in memory B cells was significantly higher in infected cases than in uninfected cases (Fig. 1b). Combined with RNA-seq analysis, we speculate one possibility that protective humoral immunity is induced to provide high affinity neutralizing antibodies for blocking virus spreading in vivo after the SARS-COV-2 attack in children.

Since the protective humoral immunity relies on the production and circulation of antibodies through the body, antigen-specific antibodies production was measured in infected children following the onset of illness. An antibody titer was performed to measure the level of antigen-specific antibodies in blood samples. Serum samples from six infected cases were collected 1–3 times as indicated after illness onset. Nucleocapsid protein and receptor binding domain of spike protein (spike-RBD) of SARS-COV-2 were selected as antigens, which were necessary for viral RNA synthesis and virus entry, respectively.^6^ Antigen-specific antibodies were detectable in five of six cases (Fig. 1c). Although one of six cases did not have antibodies for both nucleocapsid and spike-RBD protein on day 2 after illness onset, five of five cases produced total antibody and IgG antibody for both antigens around 2–3 weeks after illness onset (Fig. 1c). Relative quantitative analysis showed that total or IgG antibody for nucleocapsid and spike-RBD protein production were significantly increasing over the days after illness onset (S-Fig. 3d, 3e). According to epidemiological features among these infected children, the mean incubation period between virus exposure and symptom onset is 6.5 days,^4^ which suggests that about 3–4 weeks after first virus exposure are sufficient for these pediatric patients to produce protective humoral immunity.

Immunoglobulin class switching is a biological mechanism by which B cells switch isotopes during maturation and differentiation.^7^ However, most of the IgM antibody, especially for spike-RBD antigen, could not be detectable after illness onset (Fig. 1c), suggesting a possibility that the most of antigen-specific B cells class switching had completed within 1 week after first virus exposure. Furthermore, we selected a serum sample from one infected case, which contained a high concentration of IgG antibody for spike-RBD protein, to measure its neutralizing activity against SARS-CoV-2. As shown in Fig. 1d, the serum from the infected case could block the receptor binding between spike protein and ACE2 protein, which has been considered as the vital pathway for a virus to enter host cells and cause the infection. Pseudovirus neutralizing assay showed that serum from the infected case could neutralize SARS-CoV-2 pseudovirus (Fig. 1e). All the above data indicate that the protective antigen-specific antibodies are induced in pediatric patients and the antibodies contribute to control the virus infection.

The milder symptoms in children are striking phenomena. The underlying mechanism may promote our understanding of the spectrum of Coronavirus disease-19 (COVID-19). We investigated the characteristics of the immune response in SARS-CoV-2 infected and uninfected pediatric patients. We found that there was rapid protective antibodies production after first SARS-CoV-2 exposure and the undetected IgM antibody suggested that most of the IgM might have switched to IgG within 1 week. This efficient humoral immune response might explain why the majority of children infected with SARS-CoV-2 had milder symptoms and recovered more easily than adults. Our finding also indicates that maybe large numbers of children infected with SARS-CoV-2 are not getting “sick”. An asymptomatic child was also confirmed with ground-glass opacities in his lung and SARS-CoV-2 RNA in his sputum sample^8^ and there was the possibility of transmission of SARS-CoV-2 from asymptomatic carriers to others.^9^ However, we do not have evidence that whether the asymptomatic children can transmit the virus to others, there is still at last 1 week incubation period between first virus exposure and IgG antibody production based on our research, suggesting a risk of transmission. What is more, the monoclonal antibody therapy can be a potential therapeutic intervention for COVID-19 and vaccines-induced protective antibodies are important for the worldwide eradication of SARS-CoV-2 in the future. In summary, we reported the characteristics of the immune response in SARS-CoV-2 infected pediatric patients and found protective humoral immunity after the SARS-CoV-2 attack.

## Supplementary information

Supplemental data and materials
