# Supplementary material for: Protective humoral immunity in SARS-CoV-2 infected pediatric patients
Source: Cell Mol Immunol. 2020 May 7;17(7):768–70. doi: 10.1038/s41423-020-0438-3 (PMC7203722; doi:10.1038/s41423-020-0438-3)
Supplement: Supplementary file 1 — Supplemental data and materials [file 41423_2020_438_MOESM1_ESM.docx]

**Protective humoral immunity in** **SARS-CoV-2 infected pediatric patients**

Yaguang Zhang^*,#,1^, Jin Xu^*,#,2^, Ran Jia^*,2^, Chunyan Yi^*,1^, Wangpeng Gu^1^, Pengcheng Liu^2^, Xinran Dong^2^, Hao Zhou^3^, Bo Shang^3^, Shipeng Cheng^1^, Xiaoyu Sun^1^, Jing Ye^1^, Xuezhen Li^1^, Jia Zhang^1^, Zhiyang Ling^1^, Liyan Ma^1^, Bingbing Wu^#2^, Mei Zeng^#2^, Wenhao Zhou^#,2^, Bing Sun^#,1^

^*^Y.Z., J.X., R.J. and C.Y. contributed equally to this work.

^#^To whom correspondence may be addressed.

^1^State Key Laboratory of Cell Biology, CAS Center for Excellence in Molecular Cell Science, Shanghai Institute of Biochemistry and Cell Biology, Chinese Academy of Sciences, 320 Yueyang Road, Shanghai 200031, China;

^2^Children’s Hospital of Fudan University, 399 Wanyuan Road, Shanghai 201102, China

^3^Shanghai Kehua Bio-Engineering Co., Ltd.1189 Qinzhou Road, Shanghai 200233, China

Corresponding Author

Bing Sun: 320 Yueyang Road, Shanghai 200031, China; +86-021-54921375.

Wenhao Zhou: 399 Wanyuan Road, Shanghai 201102, China

Mei Zeng: 399 Wanyuan Road, Shanghai 201102, China

Bingbing Wu: 399 Wanyuan Road, Shanghai 201102, China

Jin Xu: 399 Wanyuan Road, Shanghai 201102, China

Yaguang Zhang: 320 Yueyang Road, Shanghai 200031, China.

Email addresses of the authors to whom correspondence should be addressed:

[bsun@sibs.ac.cn](mailto:bsun@sibs.ac.cn),

[zhouwenhao@fudan.edu.cn](mailto:wenhao.zhou@fudan.edu.cn),

[janexu@fudan.edu.cn](mailto:janexu@fudan.edu.cn),

[zengmeigao@aliyun.com](mailto:zengmeigao@aliyun.com),

[zhangyaguang@sibcb.ac.cn](mailto:zhangyaguang@sibcb.ac.cn),

bingbingwu2010@163.com

**Contributions**

B.S., W.H.Z., M.Z., B.B.W., J.X. and Y.G.Z. initiated, designed and supervised the study. Z.Y.G, B.B.W, X.R.D and S.P.C. performed RNA-seq and data analysis. Y.G.Z., J.X., R.J. and W.P.G designed and performed flow cytometry analysis. J.X., R.J., H.Z. and B.S. designed and performed antibodies titer. Y.G.Z., J.X., R.J. and C.Y.Y. designed and tested the blocking activity of serum antibodies. Y.G.Z., R.J. and C.Y.Y. performed pseudovirus neutralizing assay. P.C.L. collected the clinical data. X.Y.S, Y.J., X.Z.L, J.Z, Z.Y.L, and L.Y.M provided reagents and materials. Y.G.Z and B.S wrote the paper. The authors declare no competing interests.

**Materials and methods**

**Patients**

PBMC RNA from patient 3 was subjected to RNA sequencing for RNA-Seq profiling. Flow cytometry data analysis was based on the samples from patient 2, patient 3, patient 5 reported in Cai, J., et al.[1] and patient 6 newly confirmed using the same method described in Cai, J., et al.[1]. Serum antibody data was coming from patient 1, patient 2, patient 3, patient 4, patient 5 reported in Cai, J., et al.[1] and patient 6 newly confirmed using the same method described in Cai, J., et al.[1]. Serum from patient 1 at day 17 after illness onset was used for receptor blocking assay and pseudovirus neutralizing assay. Uninfected controls in RNA-Seq profiling and Flow cytometry data analysis were patients hospitalized during the same period without SARS-CoV-2 infection. Informed consent was obtained from the parents or guardians of the patients infected and uninfected with SARS-CoV-2 for the publication of their clinical data. Ethical approval was provided by the Hospital Ethics Committee (Ethics Approval 2020-27).

**RNA-Seq profiling in peripheral blood mononuclear cells.**

The blood sample was collected the next day after patient 3 was confirmed. Blood samples from this patient were used for transcriptome sequencing by Illumina HiSeq^TM^ 2000, paired end with 150bp. Reference transcriptome file was downloaded from GENCODE (human v33) and raw reads were directly mapped by Salmon [ref: Roberts, Adam, and Lior Pachter. “Streaming fragment assignment for real-time analysis of sequencing experiments.” Nature Methods 10.1 (2013): 71-73.]. TPM (transcript per million) was used as the expression level and the logFC (log fold change) between affected patient to the matched control was used as the differential expression profile. GSEA (gene set enrichment analysis) was performed for immune-related gene sets from MSigDB by using BP branch. ES (enrichment score), NES (normalized enrichment score) with nominal P-value was used as the statistics for significance judgment.

**Flow cytometry analysis of T and B cells.**

The cells were stained with antibodies for surface antigens in PBS with 2% FBS. All staining processes were performed according to recommended protocols. The staining was detected using an Aria II flow cytometer (BD), and the data were analyzed using Flow Jo v10 (BD). The following antibodies were used for staining: Human BD Fc Block(BD, 564220), human CD3 (BD, clone SK7), human CD4 (eBioscience, clone OKT4), human CD8 (BD, clone SK1), human CD197/CCR7 (BD, clone 3D12), human CD45RA (eBioscience, clone HI100), human CD19 (BD, clone HIB19), human CD14 (BD, clone MφP9), human CD27 (BD, clone M-T271), human IgD (BD, clone IA6-2), human IgG (BD, clone G18-145).

**An antibody titer**

A double-antigen sandwich Chemiluminescence Immunoassay (CLIA) for total antibody detection, an indirect IgG antibody CLIA, and an IgM antibody-capture CLIA were provided and performed by Shanghai Kehua Bio-Engineering Co., Ltd.

**Receptor blocking assay**

To investigate the ability of the serum from an infected case to block SARS-CoV2 Spike protein binding to ACE2, serially diluted serum (3-fold dilution from 1:10) were incubated with 150ng/ml biotinylated recombinant Spike Protein (S1+S2 extracellular domain, His tag, Sino Biological) for 1 h at 37°C. The mixture was added to plates pre-coated with 200ng of the recombinant ACE2-hFc expressed by CHO cells. After 2 h of incubation at 37°C, the wells were washed, and the Spike protein bound to ACE2 was detected with HRP-conjugated streptavidin (R&D Systems). The plates were incubated for 1 h, followed by the addition of TMB substrate. The percentage of receptor blocking activity was calculated as the reduction percentage of S binding to ACE2 compared to the value in the absence of the serum. Serum from uninfected subjects was used as a negative control.

**Pseudovirus neutralizing assay**

SARS-CoV2 pseudoviruses were produced as previously described[2]. Briefly, plasmids coding full-length spike protein and pNL4-3 luc Env- Vpr- were co-transfected into 293T cells in 10 cm dishes. The supernatants were harvested 48h after transfection and were diluted in complete DMEM mixed with an equal volume (50μl) of diluted serum and antibodies, and incubated at 37°C for 1 h. The virus-plasma mixture was transferred to 293T expressing human ACE2 stable cell line cells. The cells were incubated at 37°C for 48 h, followed by lysed with passive lysis buffer and tested for luciferase activity (Promega USA). The percent neutralization was calculated by comparing the luciferase value of serum group to those of the virus-only control.

1. Cai, J., et al., *A Case Series of children with 2019 novel coronavirus infection: clinical and epidemiological features.* Clinical Infectious Diseases, 2020.

2. Bian, C., et al., *Conserved amino acids W423 and N424 in receptor-binding domain of SARS-CoV are potential targets for therapeutic monoclonal antibody.* Virology, 2009. **383**(1): p. 39-46.

**Supplemental Figures**

**
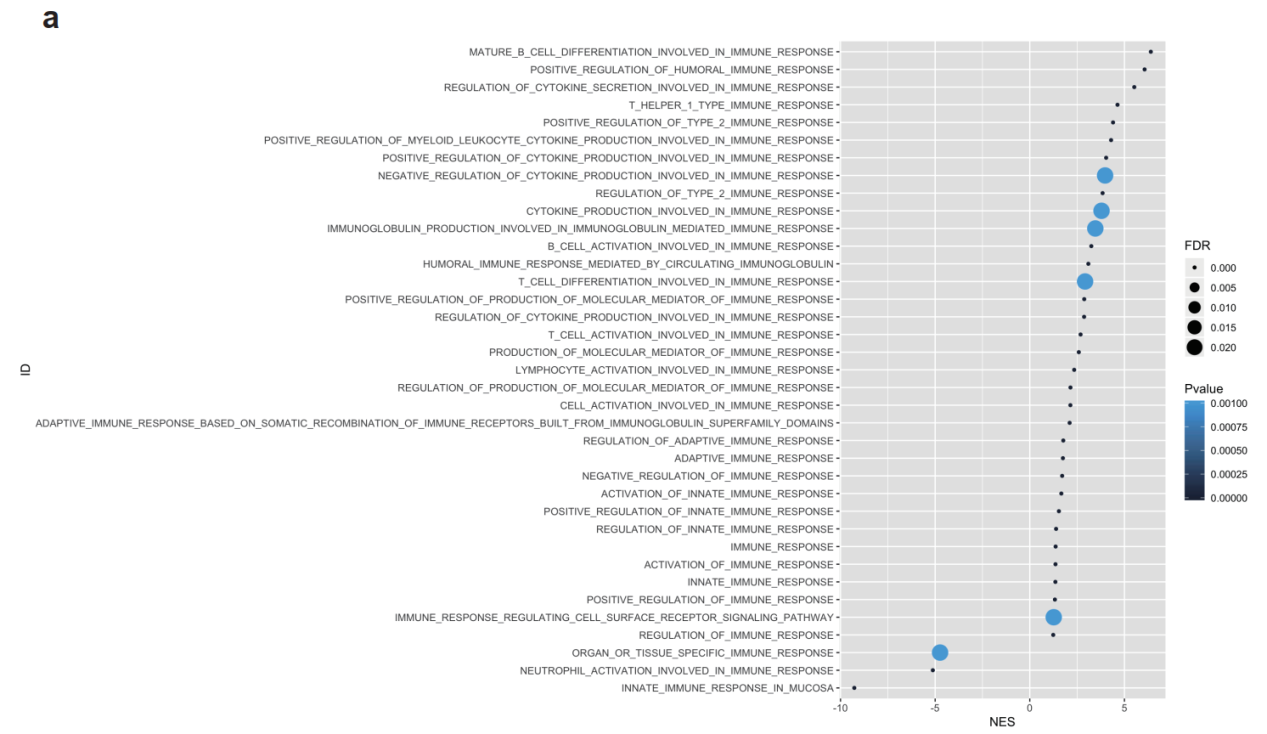
**

**S-Figure 1 RNA-Seq profiling in** **peripheral blood mononuclear cells of** **SARS-CoV-2 infected pediatric case and control.** (a) immune response-related GO categories enrichment analysis.


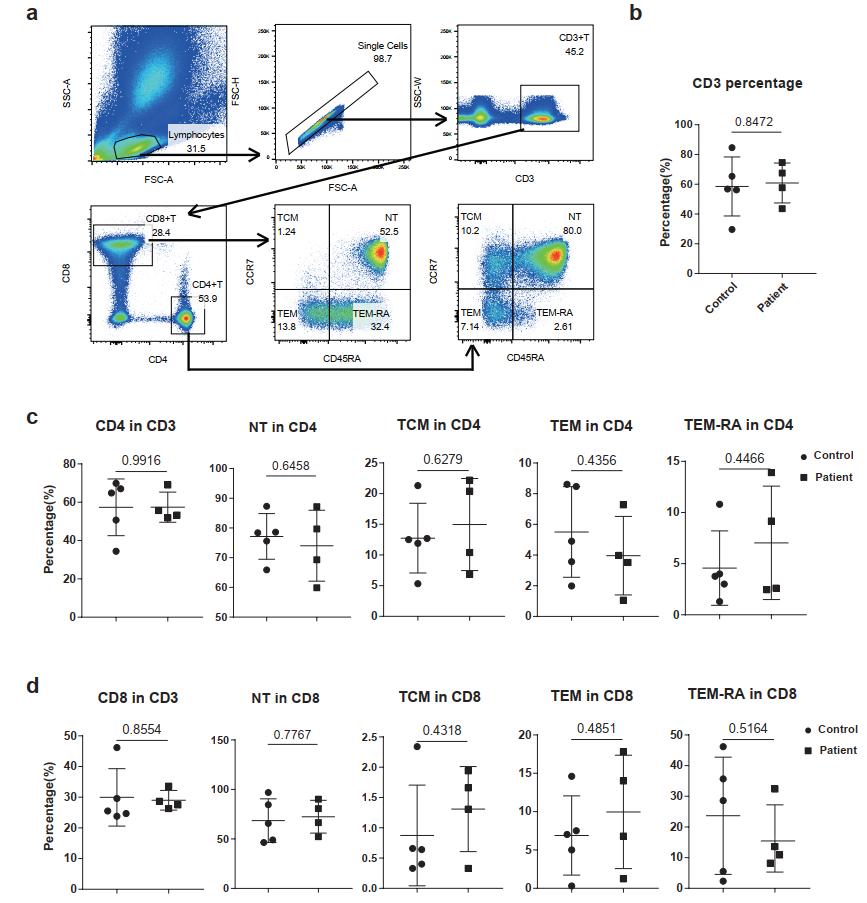


**S-Figure 2 Flow cytometry analysis of T cells in SARS-CoV-2 infected pediatric patients.** (a) T cell gating strategy. (b) CD3+ T cells percentage in single cells. (c) CD4+ percentage in CD3+ T cells, and CD45RA and CCR7 expression in CD4+ T cells. (d) CD8+ percentage in CD3+ T cells, and CD45RA and CCR7 expression in CD8+ T cells. Naïve T cell (NT: CD45RA+CCR7+), central memory T (TCM: CD45RA−CCR7+), effector memory T (TEM: CD45RA−CCR7−) and CD45RA+ effector memory T (TET-RA: CD45RA+CCR7−). The statistical method is the Student's t-test.


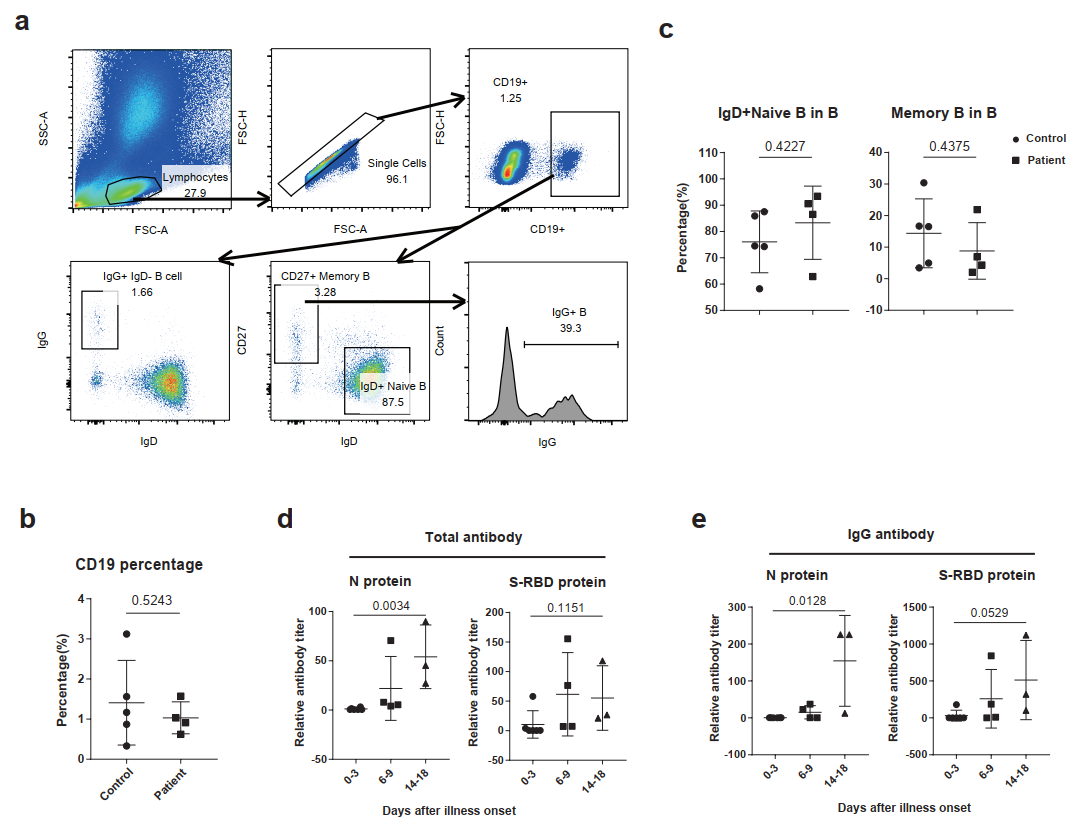


**S-Figure 3 Flow cytometry analysis of B cells and antibodies in SARS-CoV-2 infected pediatric patients.** (a) B cell gating strategy. (b) CD19+ B cells percentage in single cells. (c) Percentage of IgD+ naive B cells and CD27+ memory B cells in CD19+ B cells. Relative quantitative analysis of total antibody (d) and IgG antibody (e) production over the days after illness onset. The statistical method is the Student's t-test.
